# 

**Published:** 1906-09

**Authors:** 


### CONTENTS.

ORIGINAL COMMUNICATIONS—

Conclusions Reached from Personal Experience with Appendicitis. By Wm. Perrin Nicolson, M.D., Atlanta, Ga. .......... 361
Some New Methods in Skin Grafting. By John Egerton Cannaday, M.D., Hansford, W. Va. .... 369
Rectal Irrigation in Enterocolitis of Children. By Clarence G. Clarke, M.D., New York City. 375

EDITORIAL NOTES AND COMMENTS—

Dr. Cooper's Death .......... 378

Meat .......... 379

NEWS AND NOTES .......... 382

BOOK REVIEWS—

A Compound of Operative Gynecology. (Bainbridge and Meeker.) .......... 387
Uric Acid. (McCrudden.) .......... 388
Prophylaxis and Treatment of Internal Diseases. (Forchheimer.) .......... 388
A Non-Surgical Treatise on the Diseases of the Prostate Gland. (Overall.) .......... 389
Sanders' New Books .......... 389

CONTENTS CONTINUED .......... X

